# Impact of Salivary Amino Acid Concentrations on 8 km Running Performance in Male Undergraduate Students: A Prospective Observational Study Based on HPLC

**DOI:** 10.3390/metabo15090625

**Published:** 2025-09-19

**Authors:** Hai Zhao, Kangwei Shen, Wei Fan, Mengjie Li, Xuejun Kang

**Affiliations:** Key Laboratory of Child Development and Learning Science (Ministry of Education), School of Biological Science and Medical Engineering, Southeast University, Nanjing 211189, China; 230218247@seu.edu.cn (H.Z.);

**Keywords:** salivary amino acids, running performance, high performance liquid chromatography, 8-km run, exercise physiology

## Abstract

Purpose: To explore the potential relationship between salivary amino acid concentrations and 8 km running performance in male undergraduate students. Methods: Thirty male undergraduate students were recruited. Participants completed an 8 km run while wearing smart bracelets. Saliva samples were collected before, immediately after, and 24 h after the run. Ultra-High Performance Liquid Chromatography (UHPLC) was used to quantify salivary amino acids. Results: The fast group (average speed > 12.80 km/h) had a significantly shorter running time (35.66 ± 1.30 min, *p* < 0.001) and higher speed (13.59 ± 0.46 km/h, *p* < 0.001) than the slow group. Before the run, salivary serine concentration (20.19 µg/mL, *p* = 0.013) was higher in the fast group. After 24 h, salivary glutamine concentration (6.65 µg/mL, *p* = 0.047) was lower in the fast group. Salivary threonine concentration was positively correlated with running speed. For every 1 µg/mL increase in salivary threonine concentration, average running speed increased by 0.011 km/h, and this correlation persisted after adjusting for age and heart rate. Conclusions: This study found a positive correlation between salivary threonine and 8 km running speed, along with differences in serine and glutamine concentrations among runners with different speeds. These findings provide preliminary evidence for the relationship between salivary amino acid concentrations and running performance, though further research with larger samples and diverse exercise types is needed.

## 1. Introduction

Middle- and long-distance running events have gained immense popularity among young adults globally. Running not only serves as a way to maintain physical fitness but also as a competitive sport. Running performance is a multifaceted outcome, shaped by a complex interplay of physiological, biomechanical, psychological, environmental, and tactical elements [1]. From a physiological perspective, the traditional model emphasizes three key parameters that significantly impact running performance: maximal oxygen uptake (VO_2max_), running economy, and fractional utilization [2,3]. While these determinants can predict performance with over 95% accuracy in highly trained runners [4], in homogenous groups of distance runners, VO_2max_ values show minimal variation [5]. This indicates that there are likely other physiological factors yet to be fully explored that could contribute to differences in running performance.

Saliva, a seemingly simple bodily fluid, has a complex regulatory mechanism. Saliva secretion is intricately regulated by the sympathetic and parasympathetic innervation of the salivary glands [6]. The human body has multiple salivary glands, and the amount of saliva secreted is under the control of the cerebral cortex. Additionally, various factors such as diet, environment, age, emotions, and salivary gland diseases can modulate saliva production [7]. Saliva is a colorless and odorless fluid, with a pH ranging from 6.6 to 7.1, and it is composed of 80% water. Its organic components include mucin, mucopolysaccharides, salivary amylase, lysozyme, immunoglobulin, blood group substances, urea, uric acid, and free amino acids, while inorganic substances such as Na^+^, K^+^, Ca^2+^, Cl^−^, HCO_3_^−^, and some gaseous molecules are also present [8].

An 8 km (km) long-distance run is a significant physical stressor for the body. During such intense exercise, the body’s stress response systems are activated. One of the key stress-response axes is the hypothalamic-pituitary-adrenal (HPA) axis. When the body is exposed to the physical stress of an 8 km run, the HPA axis is stimulated. This stimulation leads to the release of stress hormones, such as cortisol [9]. These hormones can, in turn, influence the activity of the sympathetic and parasympathetic nervous systems. The sympathetic and parasympathetic nervous systems play a crucial role in regulating saliva secretion. During exercise, the sympathetic nervous system is typically activated, which may lead to a decrease in saliva production in some cases. On the other hand, the parasympathetic nervous system, which usually promotes saliva secretion at rest, may have its activity altered during running. The combined effect of the HPA axis activation and the subsequent changes in the autonomic nervous system can potentially have a profound impact on saliva secretion.

Salivary amino acids are of particular interest in this context. Amino acids are the building blocks of proteins and play essential roles in various physiological processes. Changes in salivary amino acid concentrations could reflect the body’s metabolic and physiological responses to exercise. For instance, certain amino acids may be utilized as an energy source during running, leading to a decrease in their salivary concentrations. Alternatively, the stress-induced changes in the body’s metabolism could cause an increase in the release of specific amino acids into the saliva. By measuring the salivary amino acid concentrations before and after an 8 km run, we aim to uncover the potential relationship between these changes and running performance. This study could provide novel insights into the physiological mechanisms underlying running performance. It may also have practical applications in sports training, such as using salivary amino acid profiles as a biomarker to monitor an athlete’s training status and predict their potential performance in a race.

## 2. Methods

### 2.1. Study Design

**Figure 1:**
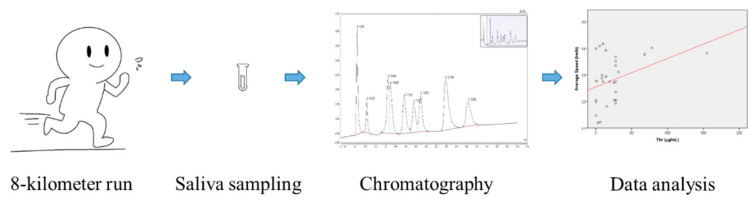
Conceptual framework of this study.

In this study, 30 male undergraduate students from Nanjing Meteorological Institute were recruited. All participants were healthy adults and were experienced at running. They were required to arrive at a specified location—a standard 400 m sports field on campus—at the scheduled time and, while wearing smart bracelets (MI Band VII, Beijing, China) provided by the research team, complete an 8 km run. These smart bracelets were capable of collecting basic exercise data, including running distance (kilometer, km), running time (minute, min), and heart rate (beats per minute, bpm). Saliva samples from participants were collected using the passive drool method. Each participant held a 2.5 mL tube under their mouth, allowing saliva to flow passively into the tube, with a collection volume of 2 mL. Saliva samples from the participants were collected three times: upon arrival, immediately after the run, and 24 h post-run. The average speed of all participants was 12.80 km/h. We defined the fast group as those with an average speed above 12.80 km/h, while the rest were considered the slow group. The study was approved by the Research Ethics Review Board of Southeast University, and written informed consent was obtained from all participants. The conceptual framework of the current study is presented in Figure 1.

### 2.2. Reagents and Chemicals

HPLC-grade methanol, HPLC-grade acetonitrile, and ethanol were purchased from Kelong Chemical Reagent, Chengdu, China. Nine amino acid standards, including alanine (Ala), arginine (Arg), aspartic acid (Asp), serine (Ser), and threonine (Thr) were from China Huixing Biochemical Reagent Co., Ltd., Nanjing, China. Glutamate (Glu), Glycine (Gly), Glutamine (Gln), and Histidine (His) were from Sigma Aldrich. Ethylenediaminetetraacetic acid disodium salt (EDTA) dehydrate was purchased from Aladdin Industrial Corporation, Shanghai, China. Citric acid and o-Phthalaldehyde were purchased from Shanghai Aladdin Biochemical Technology Co., Ltd., Shanghai, China. Sodium tetraborate and sodium sulfite were from Shanghai Titan Scientific Co., Ltd., Shanghai, China. Sodium hydrogen carbonate was from Jiangsu Yonghua Fine Chemical Co., Ltd., Nanjing, China.

The derivative reagent was prepared as follows: the ethanol solution contained one mol/L sodium sulfite (Na_2_SO_3_) and 0.4 mol/L o-Phthalaldehyde, and 0.25 mL of this solution was added to 5 mL of 0.1 mol/L sodium tetraborate (Na_2_B_4_O_7_) aqueous solution and blended thoroughly.

The mobile phase was composed of methanol/acetonitrile/water (5:6.5:88.5, *v*/*v*/*v*) containing 67 mmol/L citric acid, 103 mmol/L NaH_2_PO_4_, and 0.369 mmol/L EDTA. Mobile phase was filtered with 0.45 µm organic membranes. The flow rate, detection voltage, injection volume, and column temperature were 1 mL/min, 0.7 V, 20 µL, and 25 °C, respectively.

### 2.3. Quantification of Salivary Amino Acids

**Figure 2:**
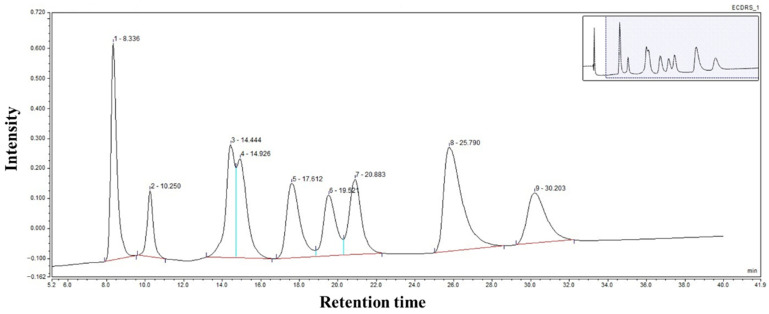
The chromatogram of the mixed standard. 1, Ser; 2, His; 3, Gln; 4, Gly; 5, Thr; 6, Asp; 7, Arg; 8, Ala; 9, Glu.

Collected saliva was first centrifuged at 12,000 rpm for 5 min in a high-speed centrifuge. Then, the supernatant was aspirated and was mixed with the aforementioned derivatizing agent for derivatization [10]. Salivary amino acids were analyzed by Ultra-High Performance Liquid Chromatography (U-HPLC) with an electrochemical detector (Ultimate 3000, Thermo Fisher, Waltham, MA, USA). The analytes were separated on a Thermo C18 chromatographic column (4.6 × 250 mm, 5 µm). The chromatograms of the mixed standards are shown in Figure 2. The linearity and accuracy of the method were assessed using seven different concentrations of amino acid standard mixtures with a linear range of 0.1–500 µg/mL, which were injected in duplicate; the recovery rates ranged from 88% to 116%. The R^2^ values of the working curves for amino acids were 0.9919–0.9982. The inter- and intra-day RSD for each amino acid were 0.39–7.59% and 0.42–7.16%, respectively. For analytes with results below the lower limit of detection (LLOD), the value was substituted with LLOD/√2 [11]. The LLODs for salivary amino acids are shown in Table 1.

### 2.4. Statistics

Means and standard deviations (SDs) were used to describe continuous variables. The independent samples *t*-test and Mann-Whitney U test were applied to describe the general characteristics and salivary amino acid concentrations among the study participants, respectively. Logistic regression was conducted to describe the associations of running pace with salivary amino acids. Two models were gradually conducted in order to adjust for covariates. Model 1 was unadjusted, providing the crude odds ratio (OR) values. Model 2 was adjusted for age and heart rate. All analyses were performed using IBM SPSS (version 22.0). A two-sided *p* value less than 0.05 was considered statistically significant.

## 3. Results

The general characteristics of the 30 undergraduate students are presented in Table 2. The running time of the fast group was 35.66 ± 1.30 min, which showed a significant difference compared with that of the slow group (39.64 ± 1.58 min) (*p* < 0.001). The average running speed of the fast group was 13.59 ± 0.46 km/h, demonstrating a significant difference when compared to that of the slow group (12.12 ± 0.53 km/h) (*p* < 0.001). In terms of age, running distance, and average heart rate, no significant differences were observed between the two groups (*p* > 0.05).

Table 3 displays the concentrations of salivary amino acids of the study participants. Before the 8 km run, the concentration of salivary serine in the fast group (median = 20.19 µg/mL) was significantly higher than that in the slow group (8.92 µg/mL, *p* = 0.013). Twenty-four hours after the run, the concentration of salivary glutamine in the fast group (6.65 µg/mL) was significantly lower than that in the slow group (24.52 µg/mL, *p* = 0.047). No significant differences were found for the remaining amino acids.

**Figure 3:**
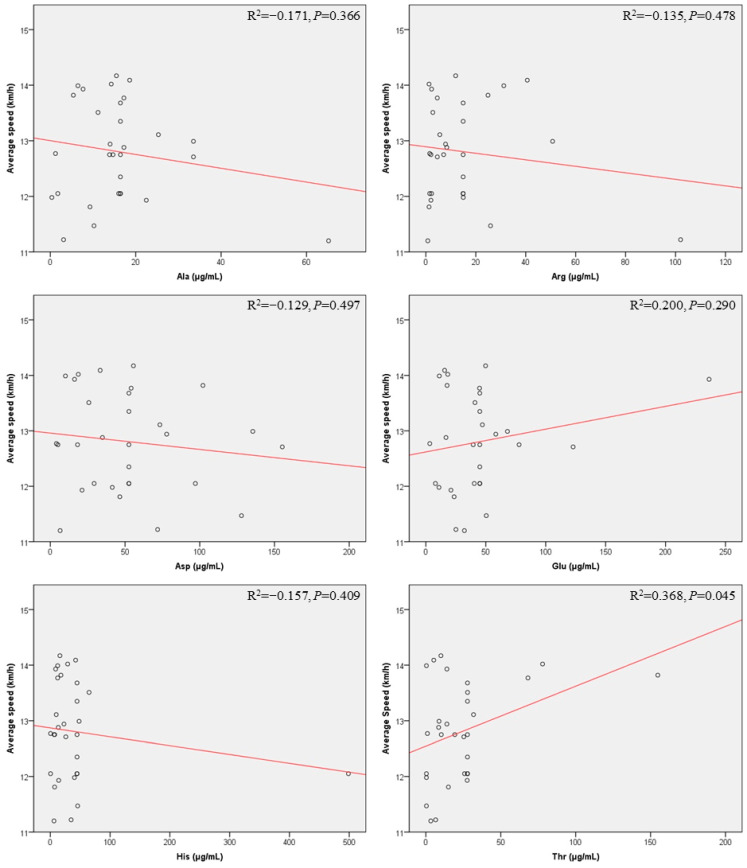
Scatter plots for correlations between salivary amino acid concentrations after run and average speed. Lines represent the linear trend. Abbreviations: Ala, alanine; Arg, arginine; Asp, aspartic acid; Glu, glutamate; His, histidine; Thr, threonine; R^2^, correlation coefficient.

Figure 3 presents scatter plots showing the relationship between the concentration of salivary amino acids after running and the average running speed. Only the amino acids with a coefficient of determination greater than 0.2 are displayed in the plot. The correlation between the concentration of serine and the average running speed was significant (*p* = 0.045). Furthermore, we conducted a linear regression analysis to explore the relationship between the concentration of salivary amino acids and running performance (Table 4). The results indicated that for every 1 µg/mL increase in the concentration of salivary threonine, the average running speed increased by 0.011 km/h, which means that the average running speed was 1.01 times (95% confidence interval (CI): 1.00–1.02) higher compared to the situation when the concentration of salivary threonine remained unchanged (*p* = 0.045). After adjusting for factors such as age and average heart rate, this correlation persisted. When the concentration of salivary threonine increased by 1 µg/mL, the average running speed was 1.01 times (95% CI: 1.00–1.02) that of the situation without the increase in salivary threonine concentration (*p* = 0.049).

## 4. Discussion

Our study focused on exploring the potential association between salivary amino acid concentrations and running performance, using a small-scale sample recruited in Nanjing. A positive correlation was observed between salivary threonine concentration and running performance. After we adjusted for age and heart rate, the association between salivary threonine concentration and running performance persisted. These findings do not imply that salivary threonine directly increases running speed; instead, they suggest that salivary threonine may act as a potential biomarker. It could reflect the metabolic changes occurring in the body when running speed improves.

There is indirect support for amino acid oxidation, since an amino acid requirement is increased after an exhaustive endurance exercise and meeting this requirement improves the recovery of performance [12]. This could indicate that amino acid oxidation was increased and that the amino acid was possibly used as an energy source. However, protein oxidation is not without consequences, as the body’s ability to store amino acids is limited, and most proteins serve critical functional roles. Consequently, understanding amino acid metabolism during endurance exercise is crucial in sports physiology. It provides insights into how the body utilizes various energy sources during prolonged physical activity and how it affects muscle recovery, adaptation, and overall athletic performance. Most amino acids undergo metabolism primarily in the liver. By contrast, branched-chain amino acids (BCAAs, including valine, leucine, and isoleucine) are believed to be metabolized in skeletal muscle—where they are also utilized as energy substrates [13]. During the degradation of BCAAs, ammonia is produced from their amino groups. Additionally, during high-intensity exercise, the forkhead box protein O1 transcription factor induces the upregulation of the glutamine synthetase gene [14]. The glutamine generated through this process is presumably transported to the liver, where it participates in the urea cycle [14]. Notably, the purine nucleotide cycle and the aspartate-malate shuttle are activated during exercise, and their metabolites further support relevant metabolic processes [15]. Collectively, these observations confirm that amino acids serve as an energy source during exercise [16].

Supplemental free-form essential amino acids (EAA) formulations promote protein synthesis and protein turnover across the entire body, with this effect extending to the synthesis of new muscle protein [17]. EAAs’ stimulation of muscle protein synthesis (MPS) is capable of promoting increases in muscle mass and quality, and these gains in turn lead to enhancements in physical performance and functional outcomes [18]. Consumption of EAA compositions stimulates MPS [19], while a decreased availability of plasma EAAs inhibits it [20]. Owing to the high intestinal absorption rate of free-form EAAs [21], swift elevations in circulating plasma EAA concentrations facilitate inward transport into muscle tissue [22], thereby leading to quicker attainment of peak intramuscular EAA concentrations compared with other dietary protein sources. The extent to which plasma EAA concentration and the rate of increase to peak concentration influence the MPS response remains unclear. However, some studies have identified an association between plasma EAA concentrations and MPS [23], and analysis of aggregated data reveals a correlation between the speed of EAA rise to peak concentrations and MPS [24].

The significant positive correlation between salivary threonine and running speed is an interesting discovery. Threonine is an essential amino acid involved in various physiological processes. During exercise, it may contribute to energy metabolism. For example, it can be converted into glucose through gluconeogenesis [25], providing an additional energy source for muscle contraction during an 8 km run. This energy supply could help maintain a higher running speed. Moreover, threonine is also a component of proteins such as collagen and elastin, which are important for maintaining the integrity and function of connective tissues in muscles and blood vessels [26]. A sufficient supply of threonine might ensure better muscle function and blood circulation during running, thereby facilitating a faster running speed.

Serine is involved in the synthesis of phospholipids [27], which are crucial for cell membrane structure and function. A higher concentration of salivary serine in the fast group might imply that these individuals have more efficient cellular functions related to energy production or muscle contraction. It could potentially reflect differences in metabolic pathways or muscle readiness for exercise. However, since the difference in serine concentration after the run was not significant, it is possible that the initial advantage in serine concentrations was consumed or metabolized during the exercise, and further research is needed to clarify its role. Glutamine is the most abundant amino acid in the body and plays a vital role in immune function, energy metabolism, and cell proliferation [28,29]. The lower glutamine concentrations in the fast group may indicate that they had a higher demand for glutamine during or after the run. Intense exercise can cause muscle damage and immune stress, and glutamine is used to repair damaged tissues and support the immune system [30]. The fast group, with their higher running speeds and potentially greater exercise intensity, might have required more glutamine for these processes, leading to a more significant decrease in its salivary concentration.

However, this study has several limitations. Firstly, the sample size was relatively small, consisting of only 30 male undergraduate students from a single university in Nanjing. A larger and more diverse sample, including different genders, ages, and fitness concentrations, is needed to generalize the findings. Secondly, only one type of exercise (an 8 km run) was investigated, and the results may not be applicable to other types of physical activities. Thirdly, although we adjusted for age and heart rate, there could still be other confounding factors, such as diet, sleep quality, and training history, which were not accounted for in the analysis.

Despite these limitations, benefiting from the convenience and non-invasiveness of saliva collection, our study provides preliminary evidence of the potential relationship between salivary amino acid concentrations and running performance. Future research should focus on increasing the sample size, conducting multi-center studies, and including a broader range of exercise types. Longitudinal and interventional studies could also be carried out to track the changes in salivary amino acid concentrations over time and their impact on running performance. This would help to further clarify the physiological mechanisms underlying the relationship and potentially lead to the development of new strategies for improving running performance in athletes.

## 5. Conclusions

This study on male undergraduates found a positive correlation between salivary threonine and 8 km running speed, along with differences in serine and glutamine concentrations among runners with different speeds. It provides valuable initial evidence, inspiring future research with larger samples and more exercise types to understand the relationship better and enhance running performance.

## Figures and Tables

**Table 1:** The lower limit of detection for salivary amino acids.

	LLOD (µg/mL)
Salivary Alanine	0.04
Salivary Arginine	0.07
Salivary Aspartic acid	0.15
Salivary Glutamate	0.09
Salivary Glutamine	0.11
Salivary Glycine	0.04
Salivary Histidine	0.10
Salivary Serine	0.07
Salivary Threonine	0.07

Abbreviations: LLOD, lower limit of detection.

**Table 2:** General characteristics of study participants.

Characteristics	Total (*n* = 30)	Slow (*n* = 16)	Fast (*n* = 14)	*p*
Age (year)	19 ± 1	19 ± 1	19 ± 1	0.387
Distance (km)	8.03 ± 0.12	7.99 ± 0.13	8.07 ± 0.09	0.084
Running time (min)	37.78 ± 2.47	39.64 ± 1.58	35.66 ± 1.30	<0.001
Average speed (km/h)	12.80 ± 0.89	12.12 ± 0.53	13.59 ± 0.46	<0.001
Heart rate (bpm)	161 ± 6	159 ± 4	163 ± 8	0.085

Note: Data are presented as mean with standard deviation (SD) for continuous variables. Abbreviations: km, kilometer; min, minute; km/h, kilometers per hour; bpm, beats per minute. *p* values were calculated using the independent samples *t*-test.

**Table 3:** Distribution of salivary amino acids among study participants.

Salivary AAs (µg/mL)	Total (*n* = 30)	Slow (*n* = 16)	Fast (*n* = 14)	*p*
Before				
Ala	8.78 (4.28, 9.28)	7.14 (3.13, 8.80)	8.91 (4.81, 10.08)	0.179
Arg	10.85 (2.95, 36.41)	5.27 (1.34, 36.41)	13.46 (7.33, 36.41)	0.294
Asp	26.54 (10.74, 64.59)	18.71 (7.46, 64.59)	29.83 (19.03, 64.59)	0.224
Glu	17.38 (11.49, 21.07)	17.38 (11.75, 19.17)	17.17 (10.76, 26.56)	0.759
Gln	28.04 (12.46, 162.85)	94.17 (14.69, 188.76)	17.30 (4.18, 162.85)	0.257
Gly	9.67 (2.07, 14.00)	8.18 (0.11, 13.18)	13.18 (2.75, 19.40)	0.166
His	11.09 (4.43, 19.87)	6.94 (2.65, 19.41)	12.14 (6.31, 19.87)	0.377
Ser	12.18 (5.89, 22.07)	8.92 (3.32,19.25)	20.19 (9.64, 29.99)	0.013
Thr	9.58 (1.44, 36.60)	9.58 (2.25, 36.60)	9.77 (1.03, 36.60)	0.667
After				
Ala	15.75 (8.89, 17.23)	15.35 (4.65, 16.42)	15.95 (10.30, 17.57)	0.473
Arg	8.13 (2.22, 14.93)	5.84 (1.65, 14.92)	10.16 (4.13, 26.46)	0.275
Asp	52.58 (20.60, 72.17)	49.55 (18.96, 66.99)	52.58 (24.01, 74.45)	0.580
Glu	42.87 (18.12, 47.80)	39.93 (21.58, 44.98)	44.88 (17.46, 51.93)	0.608
Gln	47.15 (11.89, 80.38)	38.97 (9.80, 80.38)	58.56 (21.39, 91.75)	0.854
Gly	16.01 (5.22, 24.13)	16.01 (6.39, 24.04)	17.28 (2.10, 28.46)	0.918
His	24.39 (9.64, 44.71)	30.36 (6.65, 44.71)	20.30 (12.28, 44.71)	0.667
Ser	21.29 (7.26, 34.14)	17.39 (4.69, 24.51)	24.51 (10.58, 41.23)	0.313
Thr	17.16 (6.20, 27.76)	17.16 (1.57, 27.71)	20.90 (8.71, 40.89)	0.154
24 h later				
Ala	7.51 (2.80, 19.04)	9.43 (4.48, 19.62)	4.44 (1.87, 9.37)	0.058
Arg	18.53 (6.29, 31.86)	16.75 (5.40, 26.07)	22.43 (7.33, 40.55)	0.400
Asp	8.39 (2.51, 22.66)	30.08 (14.28, 198.67)	63.70 (15.86, 106.06)	0.951
Glu	17.16 (8.69, 33.69)	29.84 (11.56, 34.61)	11.79 (4.56, 25.66)	0.101
Gln	11.51 (3.04, 31.04)	24.52 (4.37, 31.04)	6.65 (0.11, 18.68)	0.047
Gly	9.66 (2.96, 35.13)	24.83 (3.85, 41.69)	9.10 (2.29, 35.13)	0.525
His	12.02 (5.67, 15.56)	12.02 (7.08, 17.61)	10.53 (4.64, 14.58)	0.377
Ser	8.35 (4.01, 12.30)	9.67 (5.29, 13.55)	5.15 (2.17, 12.04)	0.179
Thr	8.39 (2.51, 22.66)	7.64 (2.95, 22.66)	9.39 (0.11, 22.66)	0.886

Note: Data are presented as median with the first and third quartiles (25th, 75th). Abbreviations: Ala, alanine; Arg, arginine; Asp, aspartic acid; Glu, glutamate; Gln, glutamine; Gly, glycine; His, histidine; Ser, serine; Thr, threonine; AA, amino acid. p values were calculated using Mann–Whitney U test.

**Table 4:** Regression analyses of running performance according to salivary amino acids.

	Model 1		Model 2	
	OR (95% CI)	*p*	OR (95% CI)	*p*
Speed				
Ala	0.99 (0.96–1.02)	0.366	1.00 (0.97–1.03)	0.721
Arg	0.99 (0.98–1.01)	0.478	1.00 (0.98–1.01)	0.713
Asp	1.00 (0.99–1.01)	0.497	1.00 (0.99–1.01)	0.682
Glu	1.00 (1.00–1.01)	0.290	1.00 (0.99–1.01)	0.735
Gln	1.00 (1.00–1.00)	0.738	1.00 (0.99–1.00)	0.566
Gly	1.00 (0.98–1.02)	0.993	1.00 (0.99–1.02)	0.772
His	1.00 (0.99–1.00)	0.409	1.00 (0.99–1.00)	0.421
Ser	1.00 (0.99–1.02)	0.974	1.00 (0.99–1.02)	0.799
Thr	1.01 (1.00–1.02)	0.045	1.01 (1.00–1.02)	0.049
Performance				
Ala	1.00 (0.94–1.06)	0.923	1.01 (0.95–1.08)	0.734
Arg	1.00 (0.97–1.04)	0.813	1.02 (0.98–1.07)	0.311
Asp	1.00 (0.98–1.02)	0.950	1.00 (0.98–1.02)	0.716
Glu	1.01 (0.99–1.03)	0.485	1.00 (0.97–1.03)	0.851
Gln	1.00 (0.99–1.01)	0.658	1.00 (0.99–1.01)	0.495
Gly	1.00 (0.97–1.04)	0.853	1.01 (0.97–1.05)	0.648
His	0.99 (0.98–1.01)	0.494	1.00 (0.98–1.01)	0.496
Ser	1.01 (0.98–1.04)	0.558	1.01 (0.98–1.05)	0.442
Thr	1.03 (0.99–1.08)	0.152	1.04 (0.99–1.09)	0.142

Note: Data are presented as OR with 95% CI. The slow group was used as the reference for evaluating running performance. Model 1 was unadjusted, providing the crude OR values. Model 2 was adjusted for age and heart rate. Abbreviations: Ala, alanine; Arg, arginine; Asp, aspartic acid; Glu, glutamate; Gln, glutamine; Gly, glycine; His, histidine; Ser, serine; Thr, threonine; OR, odds ratio; CI, confidence interval.

## Data Availability

The original contributions presented in the study are included in the article, and further inquiries can be directed to the corresponding author.
